# Impact and Economic Evaluation of the Patient-Provider Support Agency Model Under India’s National Tuberculosis Elimination Program: Protocol for a Cohort Study

**DOI:** 10.2196/76302

**Published:** 2026-04-06

**Authors:** Nikita Raula, Ingeborg Maria van der Putten, Nicole H T M Dukers-Muijrers, Shankar Dapkekar, Priyakanta Nayak, Silvia M A A Evers

**Affiliations:** 1 Department of Health Services Research, CAPHRI Care and Public Health Research Institute, Maastricht University, Minderbroedersberg 4-6, 6211 LH, Maastricht, P.O. Box 616 6200 MD, The Netherlands, 91 7008978561; 2 Department of Health Promotion, Care and Public Health Research Institute (CAPHRI), Maastricht University, Maastricht, The Netherlands; 3 Department of Sexual Health, Infectious Diseases and Environmental Health, Public Health Service South Limburg, Heerlen, The Netherlands; 4 Public Health Department, Disha Foundation, Nashik, India; 5 World Health Organisation Health Emergency Programme (WHE), WHO Country Office, Ankara, Turkey; 6 Centre of Economic Evaluation, Trimbos, Netherlands Institute of Mental Health and Addiction, Utrecht, The Netherlands

**Keywords:** cost-effectiveness analysis, economic evaluation, National Tuberculosis Elimination Program, Patient-Provider Support Agency, private sector engagement, quality of life, tuberculosis

## Abstract

**Background:**

India is set to eliminate tuberculosis (TB) from the country by 2025, 5 years ahead of the global target. Optimizing TB care pathways is challenged by the predominance of private sector care-seeking among symptomatic patients (60%-70%), a setting associated with significant under-notification. To improve the notification of TB among private providers and improve patient management in the private sector, the National Tuberculosis Elimination Program (NTEP), India, has implemented the Patient-Provider Support Agency (PPSA). The PPSA model enables state and district TB units to engage third-party agencies to involve private providers in TB care, providing comprehensive services including diagnosis, notification, and treatment support.

**Objective:**

The objective of this study is to assess the cost-effectiveness and feasibility of introducing the PPSA in the National TB elimination program to improve patient management by improving TB case notification, drug-resistant TB case notification, and treatment outcome and reducing loss to follow-up compared to baseline ongoing programs.

**Methods:**

A cohort study will be conducted in 2 parallel groups in the private sector to assess the impact of the PPSA intervention on the primary and secondary outcomes of the NTEP. Eligible patients with TB from the intervention and control district, notified within a defined time frame, will be included. The intervention group will receive all the facilities and benefits under the PPSA model under the NTEP. The control group will receive care under usual conditions. The study will consist of three parts: (1) impact assessment study, (2) cost-effectiveness study, and (3) feasibility and accessibility study. The primary outcomes shall be TB case notification, drug-resistant TB case notification, universal drug susceptibility testing coverage, and treatment outcome. Secondary outcomes shall include the time between the onset of symptoms and treatment initiation, treatment adherence, adverse drug reaction management, improvement in quality of life, and societal costs. Outcome assessments will be done using questionnaires at baseline, after 3 months, at the completion of treatment, and 6 months after the completion of treatment.

**Results:**

The study received Institutional ethical approval from Jodhpur School of Public Health Institutional Review Board, India, in accordance with the compliance of Section 4, National Ethical Guidelines for Biomedical and Health Research Involving Human Participants, ICMR (2017), with IRB Number: 10032/IRB/20-21. Data collection is underway.

**Conclusions:**

Our work will add to other implementation studies capturing the role of the private sector in TB care in India. A key strength of this work is its use of detailed patient pathway data from the study site, suggesting that private sector engagement can support TB elimination while improving the quality and coordination of TB services across India’s fragmented health care system.

## Introduction

### Background

Tuberculosis (TB) is caused by a bacterium called *Mycobacterium tuberculosis* and is the 13th leading cause of death and the second leading infectious cause of death after COVID-19, followed by HIV or AIDS [1-4]. About one-fourth of the world’s population is infected with TB. This can affect anyone anywhere and often affects people in their most productive years of life [5]. In 2021, 10.6 million people were estimated to be infected by TB, and 1.6 million died due to TB. India represents the highest TB burden globally, accounting for approximately 24.4% of the global TB burden [6]. The World Health Organization declared the sustainable development goals to eliminate TB globally by 2030. Universal access to TB care is key to reaching the global target of TB elimination [5]. However, not all patients receive adequate care for TB, mainly because of substantial underdiagnosis and underreporting of the disease [5]. In high-burden countries, a notable disparity exists between the number of individuals afflicted with TB and those who have been officially diagnosed and reported to the national TB programs. This incongruity highlights the need for greater vigilance and enhanced reporting mechanisms to ensure prompt diagnosis and treatment of TB cases. Failure to address this disparity could potentially transmit TB to unaffected populations and exacerbate the global burden of TB [5].

**Figure 1.**
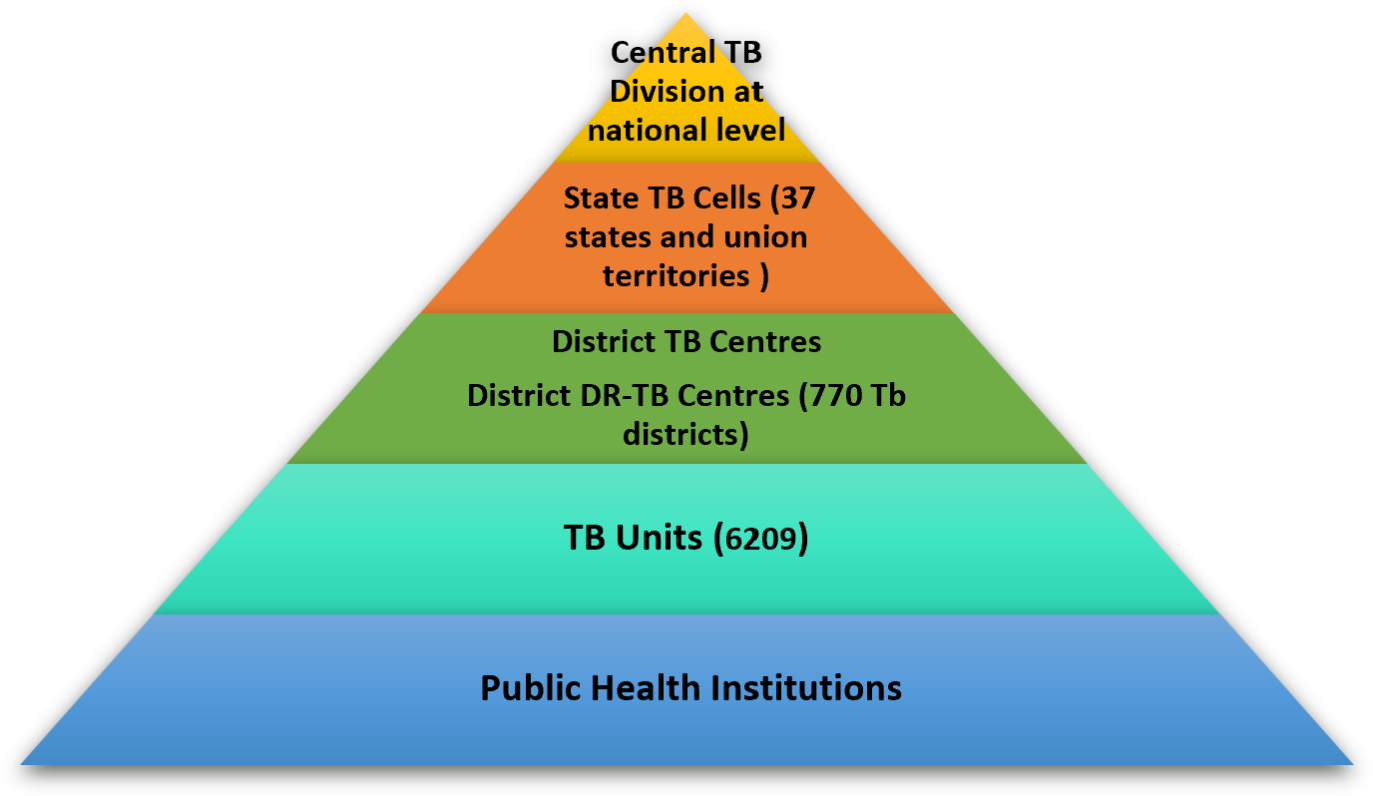
Organization of public health care response to tuberculosis (TB) in India to enable universal access to quality care. DR: drug-resistant.

Aligning with the global TB elimination goal, India launched the End TB strategy under the National Tuberculosis Elimination Program (NTEP) in 2018. The ambition is to eliminate TB from the country by 2025, 5 years ahead of the global target [6,7]. In India, 2.59 million TB cases were reported to the NTEP (incidence ~26 lakh cases) in 2021 [6]. To reduce underdiagnoses and underreporting, the NTEP has decentralized the TB services to facilitate access to TB care in the country. An overview of the organization of the TB care in India can be found in Figure 1.

A complicating factor of optimizing the pathway to managing TB care is that about 60%‐70% of the patients with TB symptoms seek care in the private health care sector [8-10]. This vast sector is highly unregulated and leading to a high rate of under-notification of cases to the national registry [11,12]. To improve the notification of TB cases among private providers, several interventions are implemented within the NTEP, such as (1) TB case notification through call centers under NTEP, (2) mapping private providers in the program’s web portal for more accessible notification, and (3) penalizing non-notification of TB. These interventions led to an increase in the notification of TB by private providers in recent years. However, private sector engagement in the program is still lagging behind, and many TB cases remain unreported from the private sector each year [5,6]. A contributing factor to the issue at hand could be insufficient awareness surrounding TB and the corresponding national TB program among private health care providers. Consequently, this has led to cases of underdiagnosis, delayed diagnoses, improper treatment of TB, as well as substandard care and support for patients with TB within the country, resulting in the emergence of drug resistance and poor treatment outcomes [13,14]. Enhancing private sector engagement in the NTEP program would be instrumental in attaining the goal of TB elimination.

To address the loopholes in the NTEP, the national government has implemented the National Strategic Plan for 2017-2025 to extend the spectrum of high-quality TB care and control, suggesting an inclusion of private sector patients in the national program (NTEP) [7]. This emphasizes the need for public-private engagement, a vital component to eliminate TB from the country. Later on, the Central TB Division conceptualized the Patient-Provider Support Agency (PPSA) in the National Strategic Plan [7]. The PPSA acts as an interface between the public and private sector care provider.

Under the PPSA model, a third-party agency is appointed by a state, city, or district NTEP unit to collaborate with private-sector physicians in providing treatment to patients suffering from TB. This model offers comprehensive services, including diagnosis, notification, patient adherence and support, and treatment linkages, thereby facilitating end-to-end care for patients with TB [15]. An agency engaged under the public-private partnership for TB care plays a pivotal role, and their responsibilities encompass a multifaceted approach to combating TB, addressing various aspects of public health and health care service coordination.

### Engagement of Private Health Care Providers

The agency’s first role involves engaging with the private health care providers, which includes mapping and prioritizing different types of private health care facilities. These encompass hospitals, clinics, charitable or trust-based institutions, as well as various other health service providers. The agency is expected to foster strong and lasting relationships with these private entities through in-person visits, workshops, and other interactions. This engagement forms a crucial link in expanding the reach of the NTEP.

### TB Case Notification

The agency is entrusted with the task of enhancing TB case notification. This involves registering private health care providers on NIKSHAY, a specialized TB case management system. The agency facilitates the generation of health establishment IDs for these providers and encourages them to directly notify TB cases. They also take the responsibility for training and supporting private providers in this notification process, ensuring that all TB cases are promptly reported.

### Sample Collection and Transportation

Effective specimen management is a critical component of the TB elimination program. The agency is responsible for the collection of specimens from private providers and their secure transportation to NTEP laboratories, whether public or private. This includes strict adherence to bio-safety precautions and ensuring proper labeling, record-keeping, and the timely delivery of test reports back to the private health care providers.

### Linkages for Free Diagnostics and Drugs

The agency is instrumental in establishing essential connections with public sector laboratories and radiology centers. This ensures that private sector patients with TB have access to free diagnostic services and radiology support. Moreover, the agency facilitates the supply of free anti-TB drugs to private patients, managing drug inventory and ensuring accurate records even in cases where patients choose to purchase drugs from the open market.

### Public Health Actions

The agency’s role expands to include various public health initiatives. This encompasses comorbidity testing for patients with TB, such as HIV and diabetes screening. It also includes universal drug susceptibility testing services, drug-resistant TB management, screening of close contacts, facilitating tuberculosis preventive treatment linkages, providing patient counseling, addressing stigma associated with TB diagnosis and treatment, and providing treatment adherence support. These actions ensure that patients with TB receive comprehensive care and support for their specific health care needs [16].

The active participation of the private sector holds the promise of notably boosting TB case reporting, aligning with national strategic plans. A study analyzing the accomplishments of PPSA in 6 states, backed by local funding, served as a compelling example of a robust and enduring collaboration between the public and private sectors contributing to increased notification of TB cases in these states. Another study involving private sector patients under the PPSA showed that free drugs increased engagement with treatment coordinators, leading to more opportunities to discuss treatment concerns, offer information, and connect with social support services. This enhanced social support likely contributes to increased treatment success [17]. These achievements provide valuable lessons for future initiatives aimed at integrating the private sector into national health programs.

As economies continue to grow and TB incidence declines, it will be increasingly challenging to maintain interest and funding for TB-only programs [18]. One possible solution to promote collaboration between individual health care providers and form networks and systems in the broader health system is the PPSA. Furthermore, the PPSA can create opportunities to include TB in other cross-cutting initiatives on health care consolidation, rationalization, and organization [18]. Introducing the PPSA can also highlight some difficult areas in the implementation of the national program and patient care that need to be addressed to develop better health systems service delivery [7]. One of these issues is dual practice, where health care workers work for both the government sector and hold a private practice. In many low-income settings in Asia, public sector salaries for health care workers are insufficient to sustain a family, leading to the need for dual practice [19].

In this research, we will assess the cost-effectiveness and feasibility of introducing the PPSA to improve patient management by enhancing TB case notification, drug-resistant TB case notification, treatment outcome, and reducing loss to follow-up under the NTEP of the Nanded Maharashtra district where the PPSA has been implemented by contracting a local agency, in comparison to care as usual under NTEP, provided in the Beed district of Maharashtra.

Previous attempts to quantify the economic and health impact of drug-sensitive TB with a shortened first-line regimen cost have been limited. These efforts have focused on general analyses that are not specific to any setting. While such studies can identify key drivers of cost-effectiveness at a general level, further work is required to characterize the cost-effectiveness of drug-sensitive TB regimens at the district level. Incorporating good empirical data on health service costs is critical [20]. One of the central aims of introducing new shortened first-line regimens is to reduce the health system burden from TB treatment [7].

Furthermore, while previous studies have included estimates of provider costs, none of these studies have considered the potential cost savings for patients. The potential benefits of patient costs are crucial in light of the global TB target of ensuring that no one suffers catastrophic expenditures from TB under universal health coverage. Through this study, we aim to focus only on drug-sensitive TB with shortened first-line regimens to include in the study group, which constitutes the majority of the TB burden. Excluding drug-resistant TB avoids methodological heterogeneity, as its care pathways are distinct and programmatically more complex.

Private sector engagement is key for TB elimination in India. Pilot studies like the Mumbai Private Provider Interface Agency and state-level PPSA implementations in Jharkhand and Odisha improved case notifications, diagnostic quality, and treatment linkage [21-24]. Despite these gains, questions remain about the long-term sustainability and economic efficiency of the PPSA. Costing studies suggest that its expenses are broadly comparable to public sector interventions, highlighting the need for further evaluation. Therefore, we will conduct a study to research the primary and secondary outcomes, the economic consequences, and the feasibility and acceptability of the patient-provider support agency intervention in the Nanded district of India.

### Objectives

To reach this overall goal, 3 studies will be conducted with the following research questions (Textbox 1).

Textbox 1. **Study framework assessing the impact, cost-effectiveness, feasibility, and acceptability of the Patient-Provider Support Agency (PPSA) under the National Tuberculosis Elimination Program (NTEP), India.**

**An Impact Study of the PPSA intervention in the district of Nanded, India**

- How does the PPSA intervention affect the primary outcomes in the NTEP in Nanded district, India?
- How does the PPSA intervention affect the secondary outcomes among the patients with tuberculosis (TB) in Nanded district, India?

**Economic evaluation of the PPSA intervention in the district of Nanded, India**

- Do the benefits of the PPSA intervention outweigh the related costs in Nanded district, India?

**Feasibility and acceptability study of the PPSA intervention in the district of Nanded, India**

- To assess the feasibility and acceptability of the PPSA intervention among the patients with TB in Nanded district, India.
- To assess the feasibility and acceptability of the PPSA intervention among the providers in Nanded district, India.

## Methods

### Design

The evaluation research will have 3 interlinked components: a cohort study, economic evaluation, and a qualitative study. The 3 studies are interlinked and together provide a broader picture of the PPSA intervention under the NTEP. The cohort study will provide the outcome data, which in turn inform the incremental cost analysis in the economic evaluation. The economic evaluation will assess the PPSA intervention from the perspectives of the health system, providers, and patients. It will include direct program costs, such as staff time, training, digital tools, monitoring, and private sector engagement, as well as indirect costs like patient time, travel, and lost income. These costs will be compared with outcomes to estimate cost-effectiveness. In line with the Medical Research Council framework, a broader cost-consequence approach will also be applied to capture a wider range of benefits, including health improvements, financial protection, and system efficiencies [25]. This will show whether the PPSA offers value for money and highlight trade-offs across sectors to guide resource allocation. The qualitative study backs the contextualization of the finding from the other 2 studies by exploring how costs and outcomes are shaped by implementation realities, stakeholder perspectives, and local health system dynamics. This triangulation will enhance the interpretation of the quantitative findings, ensuring that economic results are not viewed in isolation but in light of acceptability, feasibility, and sustainability. They form a complementary approach where the quantitative data are strengthened through the qualitative lens (Table 1).

**Table 1.** Overview of study methodology and workflow.

Category	Methodological choices
Study design	Three interlinked components: (1) cohort study on programmatic outcomes; (2) economic evaluation of cost-effectiveness; (3) qualitative study guided by Proctor’s and Fleuren’s frameworks to assess feasibility, acceptability, and sustainability of the PPSA^a^
Research settings	Conducted in Nanded (intervention) and Beed (control) districts, Maharashtra—comparable in demography and TB^b^ burden
Primary listing	Data sources: Nikshay notification registers (December 2023), private provider lists, and PPSA staff rosters
Eligibility screening	Inclusion: ≥18 years, drug-sensitive TB, residing in study districts, consent given. Exclusion: Drug-resistant TB, transfer-outs, duplicates, nonconsent
Exclusion	Noneligible, resistant cases, transfer-outs, duplicates, or refusals
Participant enrollment	Eligible participants invited, consent obtained, enrolled in database. Providers, PPSA staff, and NTEP^c^ officials included for qualitative interviews
Baseline assessment	Extraction of demographic and clinical data from Nikshay and facility records. Mapping of diagnostic and treatment pathways
Intervention	PPSA services: diagnostics, counseling, adherence support, private provider coordination, DBT^d^ benefits, and so forth
Data collection	Quantitative: baseline, 6- and 12-month follow-up surveys; cost data at 6 months quantitative interviews with the patients Qualitative: semistructured interviews (patients, providers, PPSA/NTEP staff) using Proctor’s and Fleuren’s domains
Analysis	Quantitative: compare key outcomes between sites. Qualitative: thematic analysis using Proctor (implementation outcomes) and Fleuren (determinants). Economic: incremental cost-effectiveness. Triangulation: integration of all data streams
Correlation	Strong PPSA linkage is expected to improve timeliness, adherence, and treatment success. Higher feasibility, acceptability, and enabling determinants (per Proctor and Fleuren) should correlate with sustained adoption and better outcomes

a PPSA: Patient-Provider Support Agency.

b TB: tuberculosis.

c NTEP: National Tuberculosis Elimination Program

d DBT: direct benefit transfer.

To answer research question 1, a cohort study will be conducted to assess the impact of the PPSA intervention on primary and secondary outcomes of the NTEP. The cohort will consist of all the notified cases of drug-susceptible TB from December 1 to December 31, 2023, in the private sector in the intervention district and the control district. A prestructured questionnaire will be implemented at the baseline, 3 months after initiation of the treatment, and after completion of the treatment. A follow-up will be done after 6 months of completion of the patients’ treatment.

To answer research question 2, an economic evaluation will be conducted to compare the cost-effectiveness of the PPSA in the NTEP in the intervention district and the control district with and without the intervention, respectively. Cost-effectiveness will give an overview of an increment cost to alleviate the health-related state of patients with TB getting treated at the private sector. The analysis will incorporate programmatic, intervention-specific, and patient-incurred costs, using an integrated health care and societal perspective (see Textbox 2). Quality of life (QOL) and years of life saved due to the intervention will be considered to calculate the benefit of the intervention. Each participant will be interviewed using a prestructured questionnaire.

Textbox 2. **Overview of primary and secondary outcomes (description of the outcomes is attached in Multimedia Appendix 1).**

**Primary outcome**

The primary outcome relates to 3 key elements: diagnosis, notification, and quality of care. Outcomes thus include the following:

- TB case notification
- Drug-resistant tuberculosis (TB) notification
- Universal drug susceptibility testing coverage
- Direct benefit transfer to patients
- Treatment outcome the treatment success rate among drug-sensitive TB cases

**Secondary outcome**

- The time between the onset of symptoms and treatment initiation
- Treatment adherence
- Adverse drug reaction management
- Contact tracing and isoniazid preventive therapy (IPT)
- Number of patients with TB screened for HIV
- Number of patients with TB screened for diabetes
- Improvement in quality of life of patients under treatment

**Figure 2.**
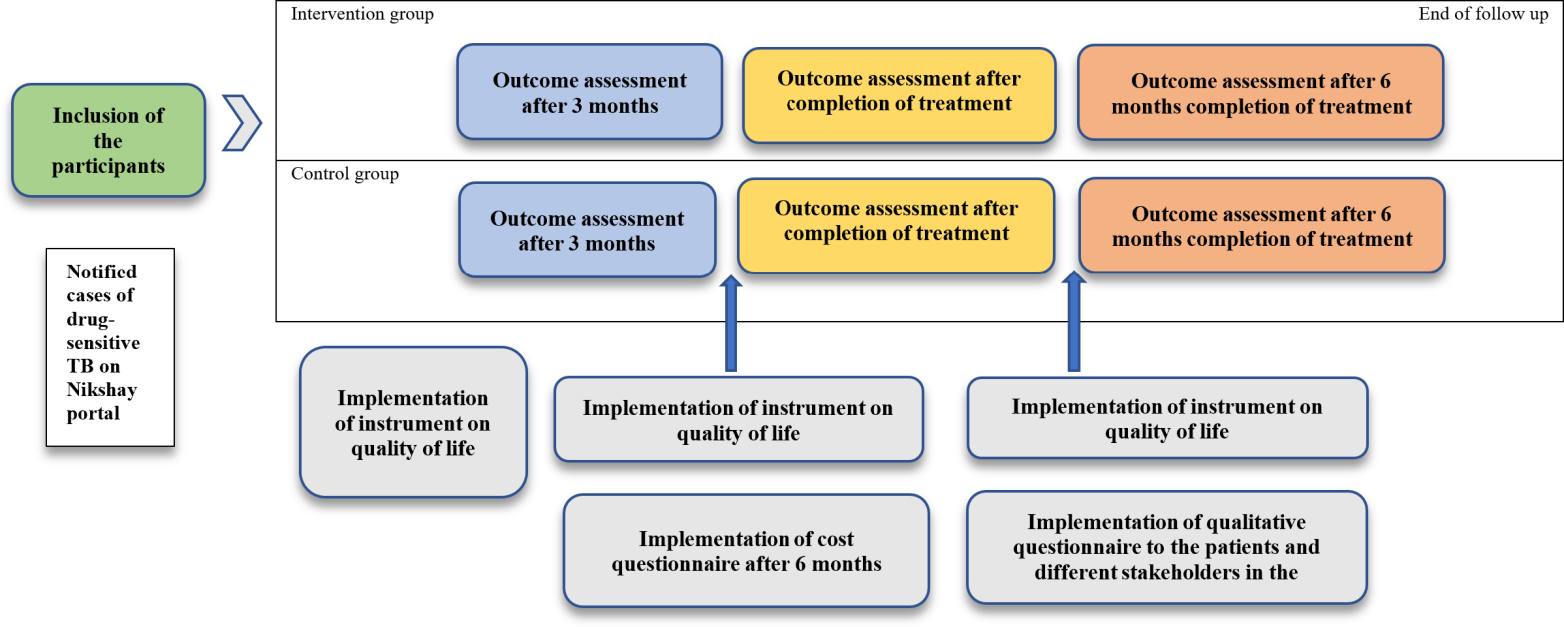
Schematic overview of the study. TB: tuberculosis.

To answer research question 3, a qualitative study using semistructured interviews will be conducted to assess the feasibility of the intervention and how that has influenced the access to TB care among the patients from the private sector and implementation challenges of the PPSA under the NTEP in Nanded district, Maharashtra, India. The schematic representation of the study framework depicting inclusion of drug-sensitive tuberculosis patients, intervention and control arms, scheduled outcome assessments at predefined time points, and administration of quality-of-life, cost, and qualitative questionnaires has been elaborated in Figure 2.

### Research Setting

Maharashtra is a large state of India with a TB notification of 2,70,000 (public sector notification 95,642 and private sector notification 64,021) in the year 2021, which is 9.4% of the total TB burden of the country [6]. The study will be conducted in Nanded district, Maharashtra, India, as the intervention district, with an area of 10,528 km^2^ and a population of 33,61,292 (rural: 2,447,394; urban: 913,898) [26]. A total of 2158 cases of TB from the public sector and 2308 cases from the private sector were notified during the year 2021 [27]. Therefore, it has many private practitioners in the urban setting who might be holding a significant proportion of patients with TB in the district. In addition, the PPSA was implemented in the district in February 2021 by the NTEP. The study will also include the Beed district of Maharashtra as a control district with comparable demography and disease burden to the intervention district. The Beed district expands to an area of 10,693 km² with a population of 2,585,049 (rural: 2,070,751; urban: 514,298) [28]. A total of 1034 cases of TB from the public sector and 862 cases from the private sector were notified during the year 2021, which is comparable to the intervention district [27].

### Study Population

All adult patients with pulmonary or extrapulmonary TB, diagnosed with microscopic slide testing or sputum culture or cartridge-based nucleic acid amplification test or clinically, who will be notified between December 1 and December 31, 2023, in the private sector in the intervention and control district, and are willing to participate in the study, will be included in the study. We will exclude the patients with confirmed drug-resistant TB, keeping in view the timeline of the study duration.

This timeframe was chosen after reviewing TB notification trends from previous months and years, which showed that notifications in December were consistent with routine patterns. Besides, the completeness of reporting and data availability with the PPSA implementation rollout was also considered to ensure the reliability of the selected cohort. While the use of a single-month sample does not capture potential seasonal variation, the aim of this study is to assess the feasibility and implementation outcomes of the PPSA model within a defined cohort. This limitation has been acknowledged, and future studies should extend to multimonth cohorts to strengthen representativeness and generalizability.

### Impact of the PPSA

In this intervention, the private providers continue managing their patients starting from diagnosis to treatment completion, along with some incentive and technical support from the program instead of transferring their patients to the public sector. Within the PPSA program, the patient receives subsidies for cartridge-based nucleic acid amplification test (Gene Xpert) test. Once diagnosed with TB, they avail the free drugs from the public sector. To help patients with treatment adherence, patients are also linked to the call center.

### Inclusion Procedures

All the patients with drug-sensitive TB notified between December 1 and December 31, 2023, in the private sector in the intervention and control districts who will be willing to participate in the study will be included in this study. After acquiring written consent, a structured questionnaire will be administered at baseline, 3 months, the completion of treatment, and 6 months after the completion of treatment.

### Measurements

The questionnaire will include demographic data of the patient and various primary and secondary outcomes listed below, along with the QOL of the patients. For the quantitative assessment, the World Health Organization *Tuberculosis Patient Cost Surveys: A Handbook* (2017) will be adapted [29]. To assess the QOL, we will be implementing a combined questionnaire of EQ-5D (EuroQol 5-Dimension Questionnaire) and SF-36 (Short Form Health Survey [36 items]) instrument, which describes various aspects of health, that is, mobility, self-care, usual activities, pain or discomfort, and anxiety or depression. The tool will be pretested among the study population to ensure appropriateness and understanding.

### Economic Evaluation

With the introduction of the new “Guidance Document on Partnerships” in late 2019, the state of Maharashtra has decided to follow output-based contracting from January 2021. According to this, PPSA implementing agencies will be paid for the program output such as notifications, the number of tests, and quality of care, instead of inputs such as incremental costs. The states must fix the cost for each service delivery according to market-based competition and technical competence of the service providers [15]. This economic evaluation will consider both health care system and societal perspectives by accounting for various costs and relating them to the incremental health-related benefits among patients with tuberculosis receiving treatment in the private sector. The intervention costs will be calculated based on the existing contract between the National Health Mission of the state and the implementing agency. A structured questionnaire will be implemented to both the intervention group and the control group to collect information on various costs incurred at the patient side, starting from the appearance of disease symptoms to completion of the treatment. Finally, the cost-effectiveness ratio will be calculated and compared between the 2 groups.

#### Qualitative Study

This qualitative study will be conducted in the intervention district (Nanded district, Maharashtra) using semistructured interviews including patients with TB, private health care providers, and program-implementing personnel. The study will be based on Proctor’s implementation outcomes framework, which provides a structure to assess implementation success through outcomes such as feasibility, acceptability, appropriateness, fidelity, penetration, and sustainability and Fleuren’s determinants of innovation for the designing and analysis to understand multilevel factors influencing implementation, such as organizational context, intervention characteristics, user-related factors, and sociopolitical influences.

The study will adopt a combination of purposive, convenience, and snowball sampling. A purposive sampling approach will be used to include key PPSA staff and relevant NTEP officials to capture context-specific insights. For the private sector, convenience sampling will be applied. Snowball sampling will be used to identify patients through practitioner referrals. Interview guides will be developed in English, translated into local languages, and will be piloted to ensure cultural appropriateness.

#### Analysis

##### Impact Analysis

Secondary data will be collected from the web portal of the NTEP and treatment registers. Baseline data will be analyzed and described. Statistical procedures will be conducted using SPSS Statistics 22.0 (IBM Corporation). Missing data will be handled using SPSS missing value analysis on item level. To calculate the impact of the PPSA, outcomes and their changes from baseline will be assessed during and after treatment completion in the intervention district and compared with those observed in the control district. The primary outcomes focus on critical aspects of TB care that reflect how well the intervention is working in real programmatic settings. These include the notification of TB and drug-resistant TB cases, and the coverage of universal drug susceptibility testing, which together indicate adherence to national standards for diagnosis and reporting. The study will also examine direct benefit transfers provided to patients through the Nikshay Poshan Yojana, as well as treatment outcomes such as cure, treatment completion, treatment failure, loss to follow-up, regimen changes, and mortality, including overall treatment success for drug-sensitive cases. Secondary outcomes will capture the essence of patient-centric care, including the time between symptom onset and treatment initiation, patient adherence as recorded in the Nikshay platform, management of adverse drug reactions, contact tracing and provision of isoniazid preventive therapy for high-risk contacts, and screening of patients with TB for HIV and diabetes. Patient-centered outcomes, such as improvements in physical, psychological, and social well-being, will also be assessed to understand the broader impact of PPSA-supported interventions on QOL. Taken together, these outcomes provide a comprehensive view of the effectiveness, coverage, and quality of TB care, allowing for a meaningful comparison between intervention and control districts under real-world program conditions. Primary and secondary outcome measures for evaluation of the PPSA intervention under the NTEP are elaborated in Textbox 2.

Baseline differences will be corrected by the inclusion of covariates in the analyses. A 2-sided significance level of .05 will be used as a threshold to determine whether differences are statistically significant. A probabilistic sensitivity analysis is planned to be conducted to examine how parameter uncertainty may impact the results of the analysis. Additionally, the combined effect of uncertainty in all parameters will be assessed [30,31]. A multivariate regression shall be used to predict missing values depending on the existing data. The precise approach will depend on the nature of the missing data: (1) missing completely at random, with no relation to the value of any other factors in the study population; (2) missing at random, but correlated in an observable way with the mechanism that generates the outcome; and (3) not missing at random, but dependent on unobserved variables [32].

##### Economic Evaluation

The economic evaluation will be performed, which will consist of a cost-effectiveness analysis [33]. Statistical procedures will be conducted using SPSS Statistics 22.0 (IBM Corporation). We intend to perform a comprehensive cost analysis considering both the health system and societal perspectives. Cost calculation will be done based on different costs incurred in the implementation of the intervention and costs from the patient perspective [34]. Intervention cost will be calculated based on the contract between the state National Health Mission and the delivering agency and partnership guidelines in Revised National Tuberculosis Control Program, India [35]. Health care and patient costs will be estimated using a questionnaire regarding health care utilization and productivity losses during the course of disease. The cost estimated in terms of rupees will be converted to US dollars [36]. The costs will be discounted over the years to make all included costs comparable. The outcome used in the cost-effectiveness analysis is the different programmatic indicators such as total diagnosed cases and treatment completion. Generic QOL is considered the main outcome, which will be calculated by comparing both EQ-5D-5L (EuroQol 5-Dimension 5-Level Questionnaire) and SF-36. Resource use and outcomes are measured at the same time points mentioned in the effectiveness study at baseline and 6 months or at the completion of the treatment in the intervention group as well as the control group. The tool to measure the QOL will be implemented at baseline, after 6 months, and after 12 months from the date of enrollment. A comparison will be made in terms of incremental costs and incremental effects. Costs and benefits included in the economic evaluation are summarized in Textbox 3.

The economic evaluation will follow the same analytical procedures as outlined for the impact analysis, including adjustment for baseline differences through covariates, application of a 2-sided significance level of .05, probabilistic sensitivity analysis to assess parameter uncertainty, and multivariate regression methods for handling missing data.

Textbox 3. **Costs and benefits included in economic evaluation.**

**A. Cost of intervention**

1. Costs of engaging private providers in the program include
  - Cost for provider training
  - Payment to the public-private mix co-ordinator
  - Subsidies to the private providers
  - Information, education, and communication material
2. Cost toward the patients
  - Subsidies to the patients for tuberculosis (TB) diagnostics or diagnostic cost (microscopy, cartridge-based nucleic acid amplification test [CBNAAT’, True NAAT line probe assay, culture, chest X-ray)
  - Cost of nutrition support to the patient
  - Cost of treatment counseling for adherence
  - Cost for the call center support mechanism
  - Cost of TB drugs
  - Sample transportation cost
  - Cost of patient house visits
  - Incentive toward transportation of the patient (max=750 Indian rupees [approximately US $8])
  - Cost of hospital stay per day

**B. Household or cost from the patient side**

 Household costs reported by the patient and the relative or guardian of the patient will be categorized into the following:

-  The cost paid to the private provider
-  Cost of drugs
-  Cost of diagnosis
-  Cost of hospital stay
-  Cost of transportation to the private provider
-  Cost of transportation to the hospital
-  Cost of transportation of the accompanying person
-  Loss of wage due to hospital visit or hospital stay
-  Loss of remuneration of the relative or accompanying person
-  Expenditure on extra food during the illness

**C. Benefits of the intervention**
  - Years of life gained
  - Quality of life

##### Qualitative Analysis

The analysis will follow these steps: (1) summarizing all the transcripts; (2) identifying units of meaning that represent different factors that are relevant to the feasibility and acceptability of the intervention among the patients and various stakeholders, and coding for these, thematically using ATLAS.ti, applying both deductive and inductive coding; (3) condensing the content of each of the coded groups; (4) integrating the insights from the condensed meaning units into generalized descriptions that reflect apparently significant factors. The Atlas software shall be used to analyze qualitative data [37]. Trustworthiness will be maintained through triangulation of data sources, member checking, reflexive journaling, and adherence to COREQ (Consolidated Criteria for Reporting Qualitative Research) reporting standards [38,39].

### Ethical Considerations

The study received Institutional ethical approval from Jodhpur School of Public Health Institutional Review Board, India, in accordance with the compliance of Section 4, National Ethical Guidelines for Biomedical and Health Research Involving Human Participants, ICMR (2017; IRB number: 10032/IRB/20‐21). Ethical written informed consent will be obtained from all the study participants. The data collection will be anonymized to ensure confidentiality. No participant compensation is planned to be provided.

## Results

The study is self-funded. As of March 2024, participant enrollment has been completed across the selected districts, and data collection is currently underway and includes patients, private healthcare providers, PPSA field staff, and program officials, using structured interviews, qualitative questionnaires, and secondary data analysis. The study aims to ensure comprehensive stakeholder representation across provider categories and implementation settings, with adequate coverage of PPSA functions, service delivery mechanisms, and associated cost components. No participant compensation is planned nor provided. Participant privacy will be protected through strict data anonymization and de-identification procedures. No personally identifiable information such as names, addresses, or contact details will be collected in the study dataset. Any reporting or publication of study findings will present aggregated results to ensure that individual participants cannot be identified.

## Discussion

Engaging with India’s vast, fragmented private health care sector is crucial in enhancing TB control in India. Our work will add to other implementation studies capturing the role of the private sector in TB care in India. A strength of this work is that it is informed by unique, detailed patient pathway data from the study site, suggesting that engaging the private sector will meet the country’s aspirations for TB elimination. Such measures would also lay the foundations for TB control by maximizing the quality and coordination of essential TB services across India’s vast and fragmented health care system.

The World Health Organization defines health as “a state of complete physical, mental and social well-being and not merely the absence of disease or infirmity.” TB is considered one of the illnesses that can seriously undermine QOL [40]. TB adversely affects all dimensions of QOL, such as general health perception, corporal sense, psychological health, mental peace, and functionality of physical and social roles [41-44]. The factors in patients affected with TB, including long-term treatment; multidrug therapy, toxic reactions and side effects of medications, adherence to drug regimen, social impacts; social support, social acceptance of the illness, family, changes in lifestyle, patients’ marital status, extent of access to health care services, socioeconomic status; patients’ and their family’s knowledge of the illness and treatments, as well as complications of TB, highly influence the QOL [22,45,46]. Still, many national TB programs across the globe universally evaluate the program success in terms of the number of people treated and cured, but not the impact of the program on well-being. Populations’ well-being needs to be focused on to improve overall productivity and economy and advocate its use as an indicator of societal progress [47]. Tools measuring QOL are most appropriate for assessing subjective well-being because they assess satisfaction and functioning that respond to life events and are relatively stable compared to substantially different, fleeting feelings of euphoria or happiness. This study will be conducted in a low- and middle-income country setting; we will use QOL for population health assessments, priority setting, and program evaluation.

There are several generic instruments that can be used across a spectrum of conditions, and Short Form 36 is commonly used in TB research, which provides the in-depth assessment of QOL among the patients with TB along 8 different dimensions [48-50]. However, while evaluating a health intervention, utility scores cannot be directly calculated from the SF-36 without taking into account the EQ-5D. The reason is that the SF-36 is not based on individual preferences, whereas the EQ-5D uses a preference-based scoring algorithm [51]. It is the most commonly used QOL assessment tool in economical evaluation and is also the most validated instrument across many countries [52].

The qualitative study adopts a multistakeholder perspective, which enhances contextual understanding. Purposive, convenience, and snowball sampling will be employed to capture diverse stakeholder perspectives, particularly from private providers and patients who are often difficult to reach through formal channels. While this approach may introduce selection bias, several strategies will be incorporated to enhance methodological rigor [53,54]. The data will be triangulated across stakeholder groups, efforts will be made to include both highly engaged and less involved providers, and reflexive documentation will be maintained throughout data collection and analysis. These measures are expected to strengthen the credibility and transferability of the findings within the operational context of the PPSA (Checklist 1) [53].

## Supplementary material

10.2196/76302 Multimedia Appendix 1 Primary and secondary outcomes [55-64].

10.2196/76302 Checklist 1 SPIRIT checklist.

## Abbreviations

COREQ: Consolidated Criteria for Reporting Qualitative Research
EQ-5D: EuroQol 5-Dimension Questionnaire
EQ-5D-5L: EuroQol 5-Dimension 5-Level Questionnaire
NTEP: National Tuberculosis Elimination Program
PPSA: Patient-Provider Support Agency
QOL: quality of life
SF-36: Short Form Health Survey (36 items)
TB: tuberculosis
