# Supplementary material for: Impact and Economic Evaluation of the Patient-Provider Support Agency Model Under India’s National Tuberculosis Elimination Program: Protocol for a Cohort Study
Source: JMIR Res Protoc. 2026 Apr 6;15:e76302. doi: 10.2196/76302 (PMC13053004; doi:10.2196/76302)
Supplement: Multimedia Appendix 1 [file resprot-v15-e76302-s001.docx]

**Multimedia Appendix 1**

**Primary outcomes:**

The primary outcome relates to three key elements, these are diagnosis, notification, and quality of care. Outcomes thus include

- TB case notification
- DR TB notification

In India, tuberculosis is a notifiable disease. Since May 2012, all healthcare providers are required to notify every diagnosed or treated TB case through the online portal Nikshay [50].

- Universal drug susceptibility testing coverage

This refers to percentage of newly diagnosed TB patients—across both public and private sectors—who receive drug susceptibility testing for rifampicin using CBNAAT (and similar technologies like TrueNat), as mandated by NTEP [51].

- Direct Benefit Transfer (DBT) to patients

Since 2018, in NTEP under Nikshay Poshan Yojana (NPY) the govt. of India provides DBT for nutritional support to all notified TB patients. Funds are transferred electronically via the Nikshay portal [52].

- Treatment outcomes[6]

Teatment outcomes for drug-susceptible TB patients

- - *Cured: Microbiologically confirmed TB patients at the beginning of treatment who was smear or culture negative at the end of the complete treatment.*
  - *Treatment completed: A TB patient who completed treatment without evidence of failure or clinical deterioration BUT with no record to show that the smear or culture results of biological specimen in the last month of treatment was negative, either because test was not done or because result is unavailable.*
  - *Treatment Success: TB patients either cured or treatment completed are accounted in treatment success.*
  - *Failure: A TB patient whose biological specimen is positive by smear or culture at end of treatment.*
  - *Failure to Respond: A case of paediatric TB who fails to have microbiological conversion to negative status or fails to respond clinically / or deteriorates after 12 weeks of compliant intensive phase shall be deemed to have failed response provided alternative diagnoses/ reasons for non-response have been ruled out.*
  - *Lost to follow up: A TB patient whose treatment was interrupted for 1 consecutive month or more.*
  - *Not Evaluated: A TB Patient for whom no treatment outcome is assigned. This includes former “transfer-out”.*
  - *Treatment Regimen Changed: A TB patient who is on first line regimen and has been diagnosed as having DRTB and switched to drug resistant TB regimen prior to being declared as failed.*
  - *Died: A patient who has died during the course of anti-TB treatment.*
- The treatment success rate among Drug Sensitive Tb cases

According to WHO, the proportion of registered TB cases those successfully completed treatment including cases that are cured or have completed treatment is called treatment success rate [6].

**Secondary outcomes:**

- The time between the onset of symptoms and treatment initiation

The interval between the initial appearance of tuberculosis-related symptoms in a patient and the commencement of appropriate anti-tuberculosis treatment [58].

- Treatment adherence

The extent to which a patient’s behavior in taking anti-tuberculosis medication corresponds with the prescribed treatment regimen in terms of timing, dosage, and duration. Adherence will be measured using the Nikshay platform, the national digital patient management system under NTEP. Nikshay records treatment initiation, drug dispensing, and follow-up data, and also integrates inputs from digital adherence technologies, providing a reliable programmatic measure of adherence [59].

- Adverse drug reaction management

The systematic identification, monitoring, and clinical management of undesirable effects caused by anti-tuberculosis drugs, aimed at minimizing harm to the patient and ensuring continuation of therapy [60].

- Contact tracing and IPT

Contact tracing refers to the process of systematically identifying, screening, and evaluating individuals who have been exposed to an infectious tuberculosis case to detect active disease or latent infection.

Isoniazid Preventive Therapy (IPT) denotes the administration of isoniazid or equivalent preventive regimens to persons at high risk of developing active TB, such as children and individuals living with HIV, to reduce future incidence [61].

- Number of TB patients screened for HIV

The proportion or count of registered tuberculosis patients who are tested for HIV infection [62].

- Number of TB patients screened for Diabetes

The number or proportion of tuberculosis patients who undergo diagnostic testing for diabetes mellitus [63].

- Improvement in Quality of life of patients under treatment

The measurable enhancement in the physical, psychological, and social well-being of tuberculosis patients as a result of treatment, encompassing symptom relief, functional recovery, reduction in stigma, and overall improvement in daily living conditions [64].
